# Anæsthesia in Dentistry

**Published:** 1879-01

**Authors:** 


### ANÆSTHESIA IN DENTISTRY.

BY DE–CE–AICH.

The question of avoiding pain in operations on the teeth and adjacent parts is frequently a difficult and vexatious problem. First of all there are those obstacles which arise from the performance of an operation in a closed cavity, difficult of access, for such is the mouth during the unconsciousness of the anæsthesics state. If the operation be a difficult one beset with contingencies, any one of which promises to overcome its efficiency, the practitioner will naturally hesitate to administer an agent which is liable to render the muscles rigid, the head helpless and the patient unable to assist. Every dentist knows that there are times when we doubt our ability successfully to perform some operations, even under the most favorable circumstances with the mouth open, a strong light, and the assistance of the patient. If now we give an anæsthetic whose operation is transient, we are not only deprived of the most favorable conditions, but we are even obliged to hasten the operation, and that under circumstances which require in the highest degree, abundant leisure for deliberate action. If on the other hand, an anæsthetic be given whose operation is of longer duration, the patient is still helpless and unable to assist intelligently. Especially if the operation is to be performed in the lower part of the mouth, the parts are deluged with blood which the patient can not remove, and which consumes the time of the operator by necessitating constant efforts to dry it up.

In view of these and other disadvantages, the magnitude and seriousness of which can not be denied, some operators decline to give anæsthetics under any circumstances. It is, however, true that anæsthetics are often of very great service. There are cases in which the above drawbacks do not exist, or still more often are capable to be overcome. There is no department of practice in which there is opportunity for more display of tact and ingenuity on the part of the dentist, than in the administration of anæsthetics, and in the per formance of operations under their influence.

